# Oncolytic Virus VV-GMCSF-Lact and Human GM-CSF Against GL261 Glioma in Immunocompetent Mice

**DOI:** 10.3390/ph19030434

**Published:** 2026-03-06

**Authors:** Alisa B. Ageenko, Natalia S. Vasileva, Anna S. Chesnokova, Dmitriy V. Semenov, Arina A. Byvakina, Maya A. Dymova, Aleksandra V. Sen’kova, Anna A. Nushtaeva, Anastasia A. Leonteva, Yulya I. Savinovskaya, Galina V. Kochneva, Vladimir A. Richter, Elena V. Kuligina

**Affiliations:** 1 Institute of Chemical Biology and Fundamental Medicine, Siberian Branch, Russian Academy of Sciences, Lavrentyev Avenue 8, Novosibirsk 630090, Russia; nataly_vas@bk.ru (N.S.V.); a.chesnokova@alumni.nsu.ru (A.S.C.); mytrilliangalaxy@gmail.com (A.A.B.); maya.a.rot@gmail.com (M.A.D.); alsenko@mail.ru (A.V.S.); nushtaeva.anna@gmail.com (A.A.N.); anastleont@mail.ru (A.A.L.); yulya_savinovskaya@mail.ru (Y.I.S.); richter@niboch.nsc.ru (V.A.R.); kuligina@1bio.ru (E.V.K.); 2 Research Center for Genetics and Life Sciences, Sirius University of Science and Technology, Krasnodar Region, Sirius (Federal Territory of Sirius), Sochi 354340, Russia; 3 State Research Center of Virology and Biotechnology “Vector”, Rospotrebnadzor, Koltsovo 630559, Russia; kochneva@vector.nsc.ru; 4 Oncostar LLC, Inzhenernaya Street 23, Novosibirsk 630090, Russia

**Keywords:** oncolytic virus VV-GMCSF-Lact, GM-CSF, glioma GL261, tumor microenvironment, transcriptome, differentially expressed genes

## Abstract

**Background/Objectives:** Oncolytic viruses are an immunotherapeutic approach that can modulate the tumor microenvironment (TME), transforming immunologically ‘cold’ tumors into ‘hot’ ones. Insertion of genes encoding immunomodulatory proteins can further enhance antitumor immune responses. In this study, we compared the antitumor and immunomodulatory effects of the double recombinant vaccinia virus VV-GMCSF-Lact, which carries the human GM-CSF gene, with those of recombinant human GM-CSF (rhGM-CSF) in an immunocompetent murine GL261 glioma model. **Methods:** The study was conducted using a subcutaneous GL261 glioma model in immunocompetent C57BL/6 mice, comparing intratumoral VV-GMCSF-Lact and rhGM-CSF treatments with evaluation of immune cell populations by flow cytometry, tumor morphology by H&E staining, and tumor transcriptome profiles by RNA sequencing. **Results:** Flow cytometry showed that VV-GMCSF-Lact reduced the number of immunosuppressive cells in the TME of subcutaneously transplanted gliomas, targeting different components of the TME depending on animal sex. The immunotherapeutic effects of rhGM-CSF were less pronounced and primarily affected peripheral immune cells. Histological analysis revealed a decrease in mitotic figures in tumors from female mice after viral therapy. Transcriptome profiling of GL261 tumors demonstrated divergent gene expression patterns and cellular compositions between treatment groups. VV-GMCSF-Lact treatment was associated with a decreased proportion of malignant GL261 cells and CD8^+^ T lymphocytes, while rhGM-CSF treatment increased proportions of MDSCs, macrophages, NK cells, and tumor-associated neutrophils. **Conclusions:** Taken together, our data demonstrate that VV-GMCSF-Lact induces antitumor immune responses in murine GL261 glioma in vivo and modulates the tumor microenvironment more effectively than rhGM-CSF alone, supporting its potential for developing new strategies for glioma treatment.

## 1. Introduction

Gliomas are the most lethal tumors of the central nervous system owing to their aggressiveness, localization in the vital brain areas, and extremely high relapse rates. The challenge of identifying efficient therapeutic strategies has been a focus of research for decades due to the marked resistance of gliomas to current treatment approaches. One of the main factors of glioma resistance is the tumor microenvironment (TME), which is characterized by a highly immunosuppressive profile and reflects host immune dysfunction. The TME is highly heterogeneous and comprises diverse tumor-associated cell populations, including tumor-associated macrophages (TAMs) and neutrophils (TANs), myeloid-derived suppressor cells (MDSCs), and regulatory T cells (Tregs). By secreting chemokines and cytokines, these cells contribute to making the tumor immunologically “cold”, i.e., effectively invisible for the immune system [1].

Oncolytic virotherapy is a potent immunotherapeutic approach that targets immunodeficient malignancies through selective immunogenic tumor cell death. It causes the release of damage-associated molecular patterns (DAMPs), pathogen-associated molecular patterns (PAMPs), and tumor-associated antigens (TAAs), thereby inducing local antitumor immune responses. DAMPs and PAMPs promote the activation of nearby dendritic cells and stimulate the release of cytokines and chemokines [2]. These factors, in turn, drive the recruitment of neutrophils and macrophages to sites of viral infection. Oncolytic viruses have also been shown to modulate TAM and TAN polarization, shifting the balance toward antitumor M1 and N1 phenotypes, respectively [3,4]. Moreover, virus-induced immunogenic cell death promotes the recruitment of CD4^+^ and CD8^+^ T lymphocytes, which contribute to the elimination of non-infected tumor cells [5].

Additional genetic modifications of oncolytic viruses can further enhance antineoplastic efficacy through the insertion of different immunomodulating or proapoptotic transgenes [6]. Arming oncolytic vaccinia viruses with GM-CSF is a widely used strategy to strengthen antitumor immunity, including in brain tumors, and it can augment immune activation beyond oncolysis alone [7,8]. At the same time, studies have shown that even GM-CSF–unarmed vaccinia virus backbones can remodel the TME and elicit both innate and adaptive antitumor immune responses [9,10]. Moreover, GM-CSF is increasingly recognized as a double-edged cytokine in cancer: it can support antitumor immunity via dendritic-cell maturation and effector T-cell activation, but it can also drive expansion and functional polarization of MDSCs and other immunosuppressive myeloid populations, particularly in gliomas [11]. Therefore, it is crucial to compare the oncolytic virus VV-GMCSF-Lact with recombinant human rhGM-CSF to test whether virus-mediated delivery provides superior antitumor efficacy relative to cytokine therapy.

Accordingly, in this work, we assessed the expression and secretion of VV-GMCSF-Lact-encoded hGM-CSF by human, mouse, and rat glioma cells infected with the oncolytic virus and investigated the effects of VV-GMCSF-Lact on the immune microenvironment of subcutaneously transplanted murine GL261 gliomas and compared these effects with those of rhGM-CSF alone.

## 2. Results

### 2.1. VV-GMCSF-Lact-Mediated Expression and Secretion of hGM-CSF by Glioma Cells

Previously, we demonstrated the high antitumor potential of VV-GMCSF-Lact against immunodeficient human glioma xenografts in vivo [12,13]. Since virotherapy is primarily an immunotherapeutic approach, the antitumor efficacy of VV-GMCSF-Lact should be evaluated in immunocompetent tumor models. It is also necessary to evaluate changes in the immune status of immunocompetent glioma-bearing mice following VV-GMCSF-Lact treatment because the virus carries transgene inserts encoding lactaptin protein and human GM-CSF, which induce tumor cell apoptosis and antitumor immune response, respectively.

In this study, we measured the level of the secreted hGM-CSF encoded by VV-GMCSF-Lact in various cell cultures: human immortalized U87MG glioma, patient-derived BR3.20 glioblastoma (grade 4), patient-derived BR5.21 anaplastic astrocytoma (grade 3), and immortalized murine (GL261 and CT2A) and rat (C6) glioma cells. Rodent glioma cells were tested in order to determine whether hGM-CSF is secreted by tumor cells after intratumoral delivery of the virus in an immunocompetent animal model.

**Figure 1:**
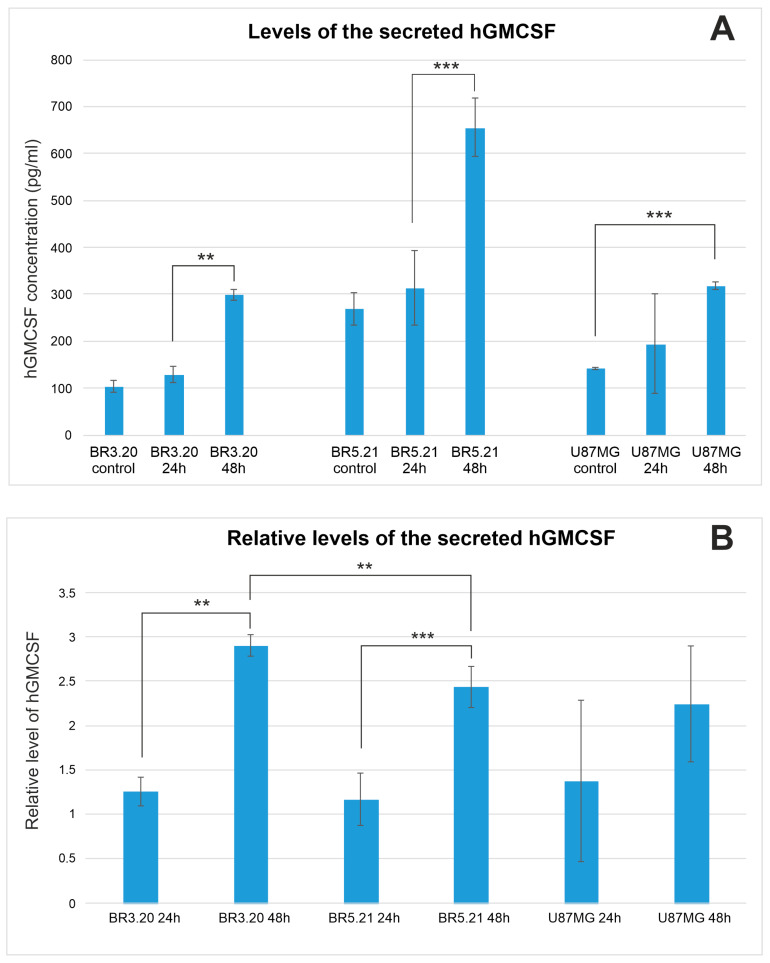
Absolute (**A**) and baseline-normalized (**B**) levels of secreted hGM-CSF in the culture medium of human glioma cells infected with VV-GMCSF-Lact. Relative hGM-CSF levels were calculated with respect to baseline cellular levels. Values represent the mean ± SD of three independent experiments (** *p* ≤ 0.01, *** *p* ≤ 0.001).

Cells were treated with the virus at the multiplicity of infection (MOI) of 0.1 plaque-forming units (PFU) per cell, and the level of hGM-CSF in the culture medium was assessed by ELISA after 24 and 48 h. In human glioma cells, secreted hGM-CSF levels increased after 48 h of incubation with the virus (Figure 1) and correlated with tumor cell sensitivity to the oncolytic activity of VV-GMCSF-Lact (Figure 1, Table 1). In contrast, hGM-CSF encoded by the oncolytic virus was not detected in the culture medium of mouse or rat glioma cells. Nevertheless, Western blot analysis demonstrated that hGM-CSF encoded by VV-GMCSF-Lact accumulated within mouse and rat glioma cells (Figure S1).

### 2.2. VV-GMCSF-Lact and rhGM-CSF Antitumor Efficacy in Immunocompetent Murine GL261 Glioma Model

**Figure 2:**
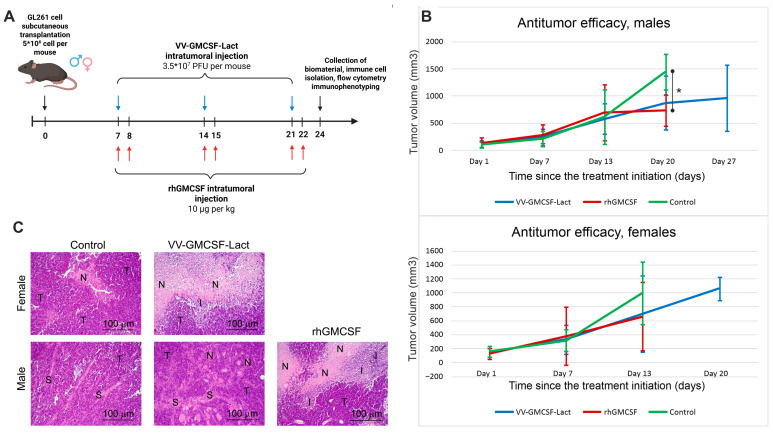
(**A**). Experimental scheme showing treatment time points (VV-GMCSF-Lact administration—blue arrows; rhGM-CSF—red arrows) following subcutaneous transplantation of GL261 glioma cells in C57Bl/6 mice. Created in BioRender. Dymova, M. (2026) https://BioRender.com/lio57nd (accessed on 15 June 2021). (**B**). Tumor volumes of male and female C57Bl/6 mice bearing subcutaneously transplanted GL261 tumors treated with VV-GMCSF-Lact and rhGM-CSF. Values represent the mean ± SD of nine (for female) and ten (for male) independent experiments; * *p* ≤ 0.05, Mann–Whitney U-test. (**C**). Structural changes in subcutaneously transplanted glioma GL261 cells in C57Bl/6 mice without treatment (control) and after administration of VV-GMCSF-Lact (male and female) or rhGM-CSF (male). Hematoxylin and eosin staining. Original magnification, ×200. T—unchanged tumor tissue; N—foci of necrosis; I—areas of infiltration; S—stromal tissue.

Although mouse and rat glioma cells appear unable to secrete hGMCSF encoded by VV-GMCSF-Lact, hGMCSF accumulates intracellularly and can be released into the extracellular space after tumor cell lysis. On this basis, we evaluated the antitumor effect of VV-GMCSF-Lact and rhGM-CSF in an immunocompetent murine GL261 glioma model established by subcutaneous transplantation. As responses to immunotherapeutic regimens differ between patients of different sexes [14,15,16], both male and female C57Bl/6 mice were included in the study. Treatment with VV-GMCSF-Lact was initiated on day 7 after tumor cell transplantation, as outlined in Figure 2A.

Tumors in male mice treated with rhGM-CSF showed a substantial reduction in volume on day 20 of therapy compared to the control group (Figure 2B). Although no significant differences in tumor volume were observed between VV-GMCSF-Lact-treated and control animals, both male and female mice from the VV-GMCSF-Lact group demonstrated the longest survival.

Histological examination of GL261 tumor nodules in control female mice revealed polymorphic atypical cells of glial origin. In the central regions of the tumor nodules, structureless necrotic areas with indistinct boundaries were detected. At the periphery of the necrotic foci, we observed mild reactive cellular infiltration at the interface with unaltered tumor tissue, consisting mainly of granulocytes with smaller numbers of lymphocytes and macrophages. A high mitotic activity was detected in the tumor tissue of control female mice, with a numerical density of mitotic cells of 7.9 ± 0.2 per field of view (Table 2).

Intratumoral injection of VV-GMCSF-Lact resulted in a marked increase in necrotic degeneration, occupying a substantial portion of the tumor nodule, and in pronounced perifocal granulocyte infiltration in female mice (Figure 2C). In addition, we observed a 1.4-fold decrease in the number of mitotic figures in tumor tissue compared to the control group (Table 2).

In control male mice, GL261 tumor nodules were also composed of polymorphic atypical cells of glial origin but had a honeycomb-like structure due to a prominent stromal component. Necrotic degeneration in the central region of the tumor nodules was mild, and mitotic activity was low (2.7 ± 0.6 mitotic cells per field of view; Table 2).

Intratumoral injection of the oncolytic virus induced small, round-shaped, diffuse necrotic foci distributed throughout the tumor tissue (Figure 2C). The mitotic cell density did not differ between control males and VV-GMCSF-Lact-treated males (Table 2). However, intratumoral administration of rhGM-CSF resulted in a near-complete necrotic degradation of the tumor tissue, although the mitotic cell density remained comparable to that in the control and VV-GMCSF-Lact groups. Pronounced neutrophilic infiltration was observed at the interface between necrotic foci and unaltered tumor tissue.

### 2.3. Immune Status of Tumor-Bearing C57Bl/6 Mice After Therapy with VV-GMCSF-Lact and rhGM-CSF

Oncolytic viruses can modulate the TME, converting immunologically “cold” tumors into “hot” ones by inducing immunogenic tumor cell death [17]. GM-CSF, on the other hand, can exert both antitumor and protumor effects. Its antitumor activity is associated with increased production of neutrophils and proinflammatory cytokines and activation of CD4^+^ and CD8^+^ T lymphocytes, whereas its protumor activity involves regulation of the TME and expansion of immunosuppressive cell populations [11]. Taking into account the potential dual immunotherapeutic effects of the oncolytic vaccinia virus VV-GMCSF-Lact, we used flow cytometry to assess the composition and number of immune cells (T and B lymphocytes, FoxP3^+^ Tregs, MDSCs, and M1/M2 macrophages) in tumors, spleens, and blood from male and female C57Bl/6 mice bearing subcutaneously transplanted GL261 tumors, with and without VV-GMCSF-Lact or rhGM-CSF treatment. Healthy, tumor-free mice were used as intact controls.

**Figure 3:**
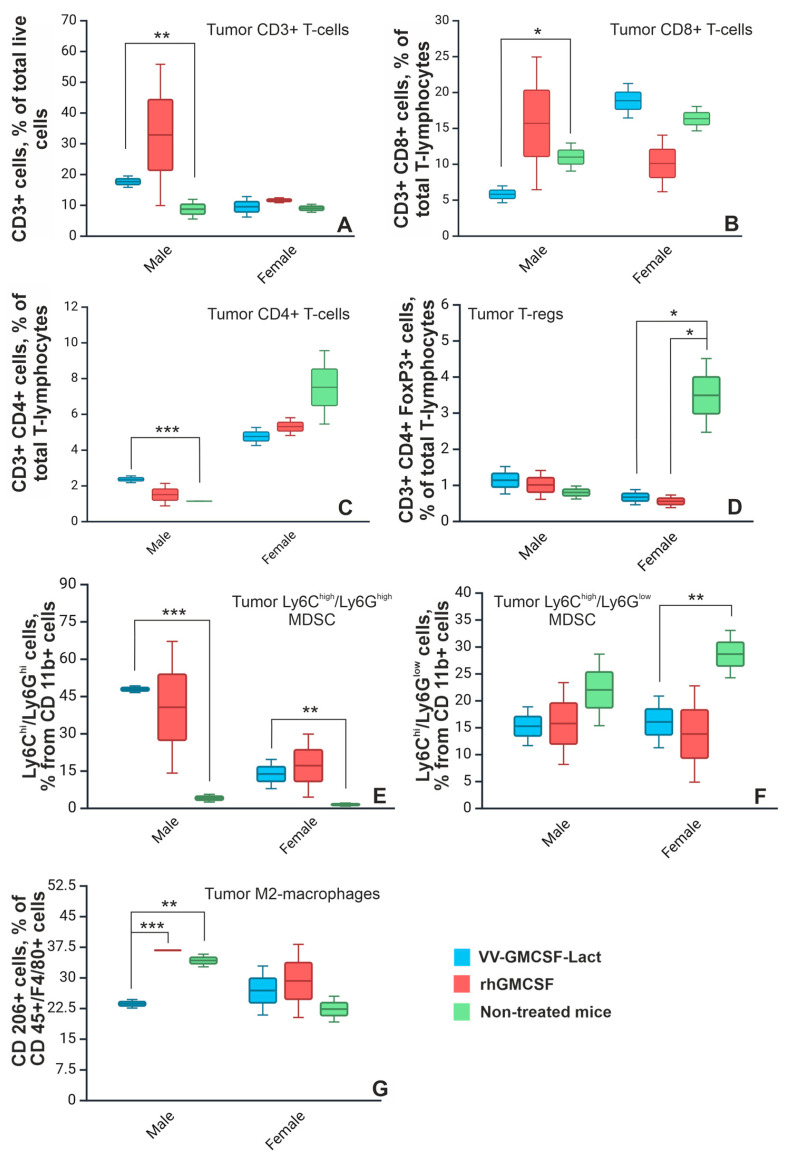
Tumor-infiltrating CD3^+^ (**A**), CD8^+^ (**B**), CD4^+^ (**C**), FoxP3^+^ (**D**) T-cell populations, myeloid-derived suppressor cells (MDSC) populations (**E**,**F**), and macrophages (**G**) in subcutaneously transplanted GL261 tumors after VV-GMCSF-Lact and rhGM-CSF therapy. Values represent the mean ± SD of three independent experiments (* *p* ≤ 0.05, ** *p* ≤ 0.01, *** *p* ≤ 0.001).

Analysis of the data revealed sex-dependent differences in tumor-infiltrating immune cells. After VV-GMCSF-Lact therapy, tumors from male mice were characterized by increased numbers of CD3^+^ and CD4^+^ T cells and a decreased number of CD8^+^ T cells compared to the non-treated control group. No differences in the number of tumor-infiltrating FoxP3^+^ Tregs were observed between treated and non-treated male mice. In female mice, no significant changes were detected in the numbers of tumor-infiltrating CD3^+^, CD4^+^, and CD8^+^ T cells compared with untreated controls (Figure 3A–C). However, the number of FoxP3^+^ Tregs decreased after both VV-GMCSF-Lact and rhGM-CSF treatment (Figure 3D).

Immunophenotyping of CD11b^+^ MDSCs identified two major subpopulations: Ly6C^high^/Ly6G^low^ cells, or monocytic MDSCs, and Ly6C^high^/Ly6G^high^ cells, which are presumed to be neutrophils and will be discussed below. We observed increased numbers of Ly6C^high^/Ly6G^high^ neutrophil MDSCs in the tumors from both male and female mice treated with VV-GMCSF-Lact compared to the respective non-treated control groups (Figure 3E). Ly6C-positive cells are known to contribute to the proinflammatory status of the tissues they infiltrate [18]. Analysis of monocytic MDSCs showed that, in female mice, the number of Ly6C-positive tumor-infiltrating cells was significantly lower following VV-GMCSF-Lact treatment compared with non-treated controls (Figure 3F).

Analysis of the macrophage population indicated that F4/80^+^ cells consisted exclusively of a CD206^+^ M2-polarized macrophage population, which is characterized by an immunosuppressive phenotype [19]. In male mice, the number of tumor-infiltrating CD206^+^ cells was significantly lower in the VV-GMCSF-Lact-treated group than in both rhGM-CSF-treated mice and non-treated tumor-bearing control mice (Figure 3G).

Peripheral lymphocyte populations in the spleen and blood were also altered after treatment of tumor-bearing animals. In male mice, VV-GMCSF-Lact administration led to a decrease in CD3^+^ and CD8^+^ cells and FoxP3^+^ Tregs in the spleen compared with the rhGM-CSF-treated, untreated, and intact groups (Figure S2A,F). In contrast, rhGM-CSF therapy increased the numbers of CD8^+^ and FoxP3^+^ T cells in the spleen relative to both the VV-GMCSF-Lact and untreated groups (Figure S2C,F).

In female tumor-bearing mice, oncolytic virus therapy reduced the numbers of CD3^+^, CD4^+^, and CD19^+^ lymphocytes in the spleen compared to intact animals (Figure S2A,D,E). Moreover, both VV-GMCSF-Lact and rhGM-CSF therapies boosted the number of CD8^+^ lymphocytes in the spleen compared to non-treated tumor-bearing mice and intact group, with the effect being more pronounced in the rhGM-CSF group (Figure S2C).

Additionally, rhGM-CSF increased the number of CD3^+^ T cells in the blood of both sexes compared to intact animals (Figure S2B).

### 2.4. Differential Gene Expression in Transplanted GL261 Glioma Tumors Treated with VV-GMCSF-Lact and rhGM-CSF

**Figure 4:**
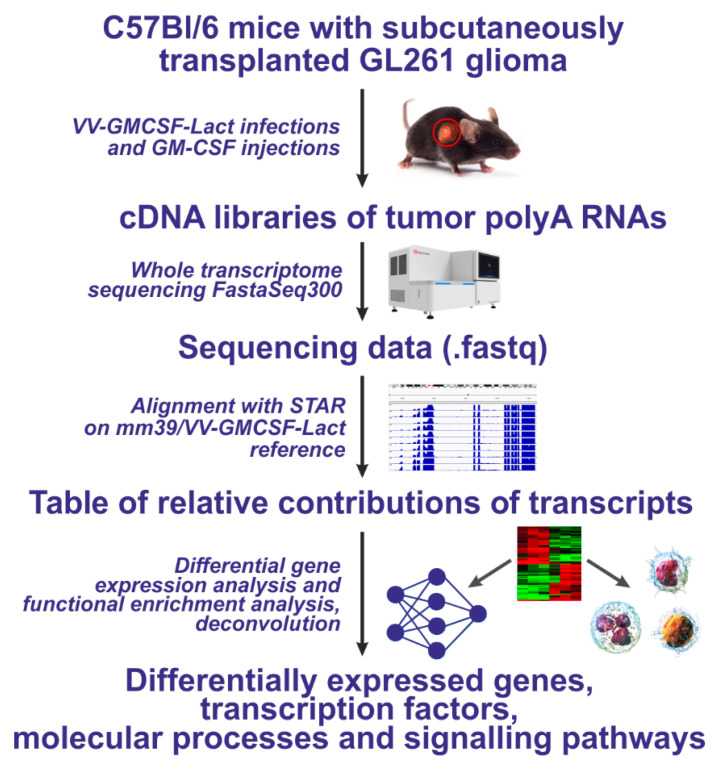
Experimental workflow for transcriptome analysis of subcutaneously transplanted GL261 tumors in C57Bl/6 mice treated with VV-GMCSF-Lact or rhGM-CSF.

To assess how the oncolytic virus VV-GMCSF-Lact and rhGM-CSF affect the tumor transcriptome, we transplanted murine GL261 glioma cells into immunocompetent C57Bl/6 mice. The established tumors were treated by intratumoral injection of VV-GMCSF-Lact or rhGM-CSF. After completing therapy, the tumors were removed, and total RNA was isolated from tumor tissue (Section 4.4). We then prepared whole-transcriptome cDNA libraries from the polyA fraction of total tumor RNA and performed high-throughput sequencing (Figure 4).

Raw high-throughput sequencing data contained between 1.4 × 10^7^ and 3.1 × 10^7^ reads (Table S1). The data were obtained for mice of both sexes; however, in this study, we combined the groups and did not stratify the transcriptome analysis by sex.

**Figure 5:**
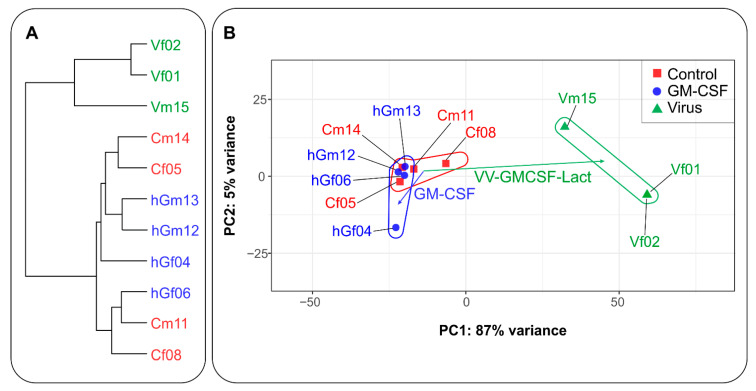
Relationships between transcriptomes of GL261 tumors subcutaneously transplanted into C57Bl/6 mice, visualized by hierarchical clustering (**A**) and principal component analysis (**B**). Samples include VV-GMCSF-Lact-infected tumors (Vf/m), rhGM-CSF-treated tumors (hGf/m), and control tumors (Cm/f). Sample designations are listed in Table S1. Arrows indicate vectors representing changes in the averaged PC1:PC2 coordinates.

The data presented in Figure 5 show that VV-GMCSF-Lact produced a larger absolute shift in PC1:PC2 coordinates than rhGM-CSF. By contrast, rhGM-CSF injections resulted in distinct but more limited changes in PC1:PC2 coordinates of tumor transcriptomes (Figure 5B). The principal component analysis was supported by hierarchical clustering (Figure 5A). Transcriptomes from VV-GMCSF-Lact-infected tumors formed a separate branch of the hierarchical tree, whereas transcriptomes from control and rhGM-CSF-injected tumors clustered together, indicating similar RNA expression patterns.

#### 2.4.1. Differential Gene Expression in GL261 Tumors Treated with VV-GMCSF-Lact

**Figure 6:**
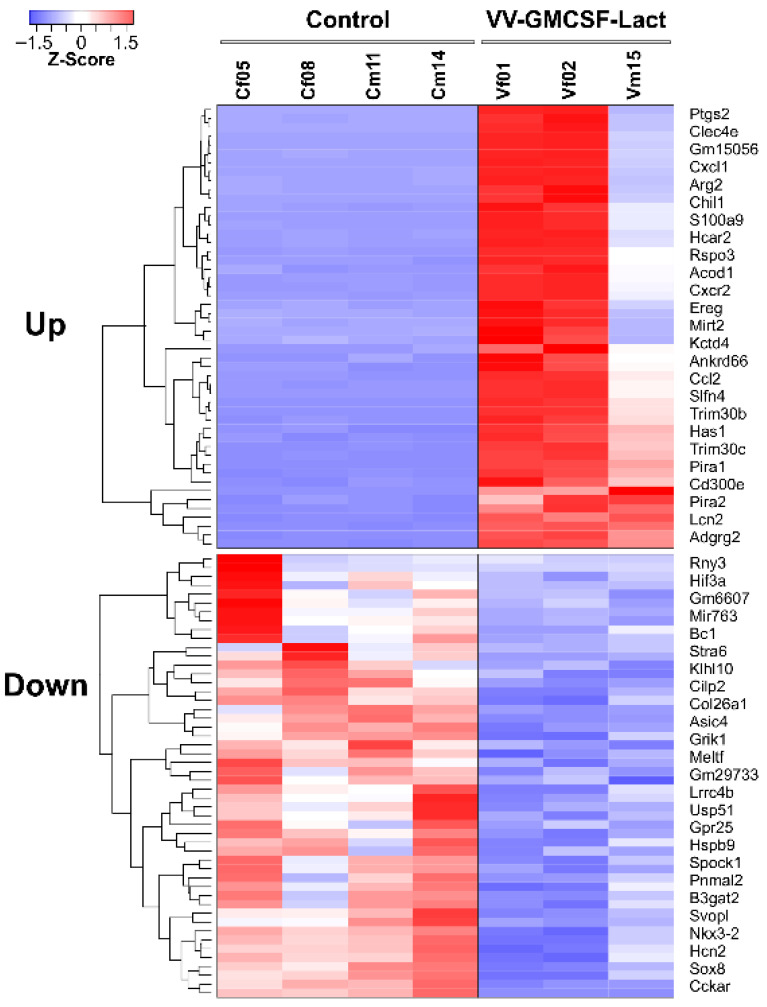
Heat maps of gene expression changes in subcutaneously transplanted GL261 tumors following VV-GMCSF-Lact infection in C57Bl/6 mice. Normalized expression levels of the top 50 transcripts that were upregulated or downregulated in response to VV-GMCSF-Lact infection of tumors are shown. Differential gene expression analysis was performed using DESeq2 (v1.36.0).

To analyze the effects of VV-GMCSF-Lact on molecular processes in GL261 tumors, we identified differentially expressed transcripts using DeSeq2 (Figure 6).

**Figure 7:**
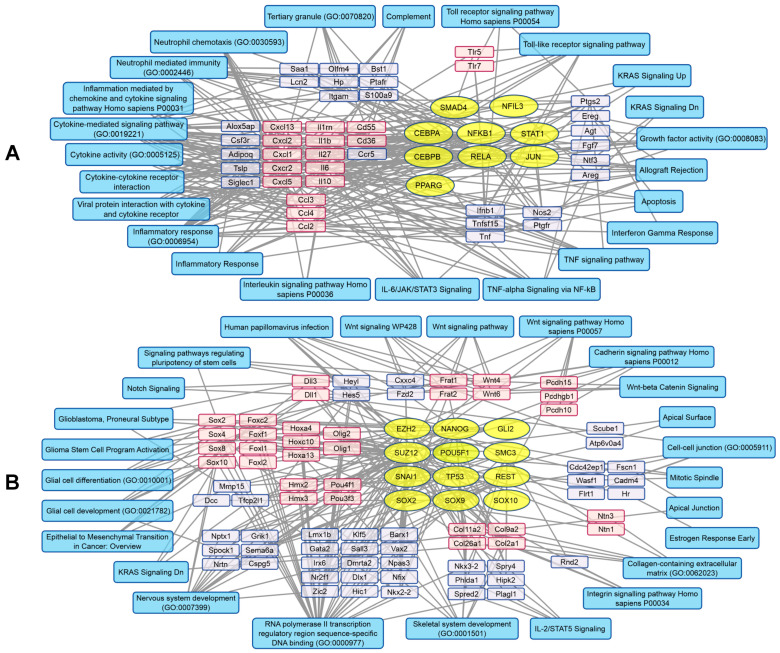
Transcription factor regulation in GL261 tumors during VV-GMCSF-Lact infection. Transcription factors are shown as yellow ovals, mRNA transcripts as blue and pink rectangles, and major biological processes involving proteins encoded by the corresponding genes as cyan rectangles. (**A**) Upregulated genes and associated transcription factor activation. (**B**) Downregulated genes and associated transcription factor suppression. Groups of functionally related genes are clustered and shown in pink. The analysis was based on the top 300 correlated genes using Enrichr libraries: ENCODE and ChEA Consensus transcription factors from ChIP-X, MSigDB Hallmark 2020, GO Biological Process 2021, Panther 2016, and KEGG 2021.

We analyzed biological processes associated with genes whose expression increased or decreased during viral infection using gene set enrichment analysis with Enrichr via the R package enrichR (v3.4) [20]. VV-GMCSF-Lact infection activated several transcription factors, including NFKB1/RELA, STAT1, CEBPA/CEBPB, and JUN (Figure 7A). At the same time, the relative level of mRNAs encoding chemokines (Cxcl1, -2, -5, and -13), interferon Ifnb1, and multiple interleukins (Il6, -10, -27, -1b, -1rn) increased. These transcriptional changes corresponded to activation of inflammatory and immune responses, cytokine signaling pathways, responses to interferon-γ, TLR signaling pathways, and neutrophil chemotaxis (Figure 7A).

VV-GMCSF-Lact infection suppressed the activity of several transcription factors, including SOX2, -9, -10, SUZ12, and TP53 (Figure 7B). Simultaneously, it reduced the relative levels of transcripts encoding SOX family members (Sox2, -4, -8, -10), forkhead box proteins (Foxc2, -f1, -l1, -l2), cadherins of the protocadherin superfamily (Pcdh15, -g1d, -h10), and collagens (Col2a1, -11a2, -26a1, -9a2). These changes are associated with suppression of integrin-, collagen-, and cadherin-mediated signaling, the Wnt signaling pathway, and a number of other processes. Importantly, the cellular response to infection included suppression of epithelial–mesenchymal transition, reduced expression of genes characteristic of the proneural glioblastoma subtype, and attenuation of pathways linked to glioma cell activation and development (Figure 7B).

Together, these changes in gene expression patterns indicate that VV-GMCSF-Lact induces cellular immune response to viral infection in transplanted glioma tumors while simultaneously suppressing molecular processes associated with malignant tumor growth.

#### 2.4.2. Differential Gene Expression in GL261 Tumors Treated with rhGM-CSF

Since the rhGM-CSF gene is integrated into the genome of the oncolytic virus VV-GMCSF-Lact, it is important to compare the effects of viral infection and rhGM-CSF administration on biological processes in GL261 tumors. As shown in Figure S3, injection of rhGM-CSF modulated the expression of multiple mRNAs.

**Figure 8:**
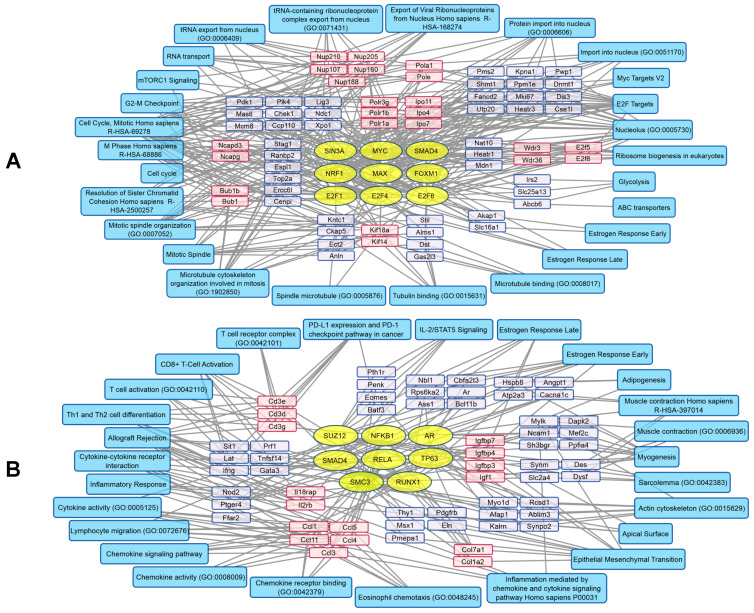
Transcription factor regulation in GL261 tumors after rhGM-CSF injection. Transcription factors are shown as yellow ovals, mRNA transcripts as blue and pink rectangles, and major biological processes involving proteins encoded by the corresponding genes as cyan rectangles. (**A**) Upregulated genes and associated transcription factor activation. (**B**) Downregulated genes and associated transcription factor suppression. Groups of functionally related genes are clustered and shown in pink. The analysis was based on the top 300 correlated genes using Enrichr libraries: ENCODE and ChEA Consensus transcription factors from ChIP-X, MSigDB Hallmark 2020, GO Biological Process 2021, Panther 2016, and KEGG 2021.

rhGM-CSF treatment activated several transcription factor families, including E2F (E2F1, -4, -6), SMAD4, FOXM1, and MYC/MAX, among others (Figure 8A). This transcription factor activation was accompanied by increased expression of genes encoding RNA polymerase subunits (Polr1a, -1b, -3g) and nuclear import proteins (Ipo4, -7, -11). We also observed upregulation of transcripts encoding the marker of proliferation Mki67, mitotic checkpoint regulators (Bub1, -1b), and members of the nucleoporin family (Nup107, -160, -188, -205, -210), which mediate RNA and protein transport through nuclear pores. In addition, rhGM-CSF activated processes associated with microtubule organization (Kif4a, -11), mitotic spindle assembly, and regulation of the cell cycle and cellular metabolism via the mTORC1 signaling pathway (Figure 8A).

rhGM-CSF also decreased the activity of several transcription factors, including NFKB1, RELA, SUZ12, and SMAD4 among others (Figure 8B). This led to suppression of transcripts linked to cytokine responses and, more broadly, the NFKB1/RELA-controlled immune response. Genes encoding insulin-like growth factor binding proteins (Igfbp3, -4, -7), T-cell receptor components (Cd3d, -e, -g), collagens (Col7a1, -1a2), and CC-chemokines (Ccl1, -3, -4, -5, -11) were downregulated as well. Notably, processes related to the immune checkpoint inhibitor PD-L1, allograft rejection, and cytokine signaling were suppressed (Figure 8B).

**Figure 9:**
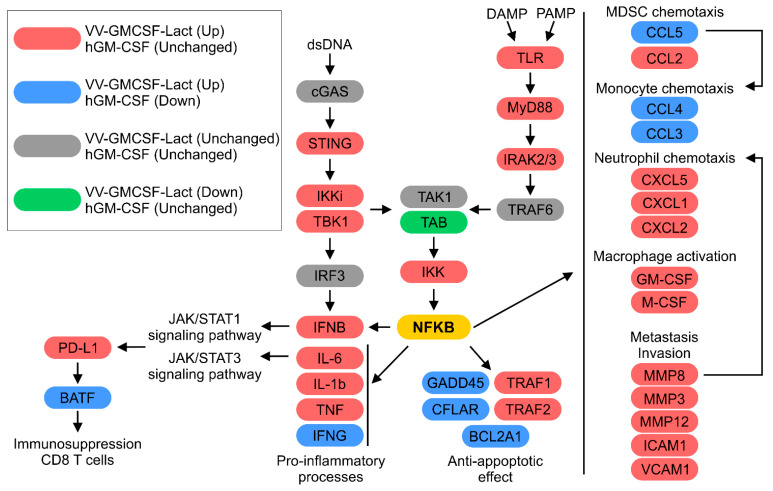
Activation of NFKB-associated signaling pathways in GL261 tumors following VV-GMCSF-Lact infection or rhGM-CSF injection. VV-GMCSF-Lact and hGM-CSF indicate differentially expressed genes after VV-GMCSF-Lact therapy and rhGM-CSF therapy, respectively. Up—genes with increased expression; Down—genes with decreased expression; Unchanged—genes without detectable differential expression.

Thus, VV-GMCSF-Lact and rhGM-CSF produced divergent effects on NFKB-regulated signaling pathways, as summarized in Figure 9. Transcriptomic analysis revealed that VV-GMCSF-Lact therapy upregulated key components of the cGAS-STING and TLR signaling pathways, including *Sting*, *Ikki*, *Tbk1*, *Tlr*, *Myd88*, and *Irak2/3*, whereas rhGM-CSF treatment had minimal impact on these genes. Viral infection also activated NFKB, upregulated NFKB-associated genes, and stimulated expression of Pdl1 and the transcription factor BATF. In contrast, rhGM-CSF reduced BATF mRNA levels without affecting *Pdl1*. Additionally, VV-GMCSF-Lact strongly upregulated proinflammatory cytokines (*Ifng*) and anti-apoptotic genes (*Gadd45*, *Bcl2a1*, *Cflar*), while rhGM-CSF induced opposing effects. Gene expression of chemokines associated with immune cell recruitment (Ccl3, -4, -5) increased with viral therapy but decreased after rhGM-CSF treatment. These findings indicate that VV-GMCSF-Lact and rhGM-CSF exert distinct and often opposing effects on NFKB signaling and immune modulation within the TME (Figure 9).

#### 2.4.3. Cellular Composition of GL261 Tumors Treated with VV-GMCSF-Lact or rhGM-CSF

**Figure 10:**
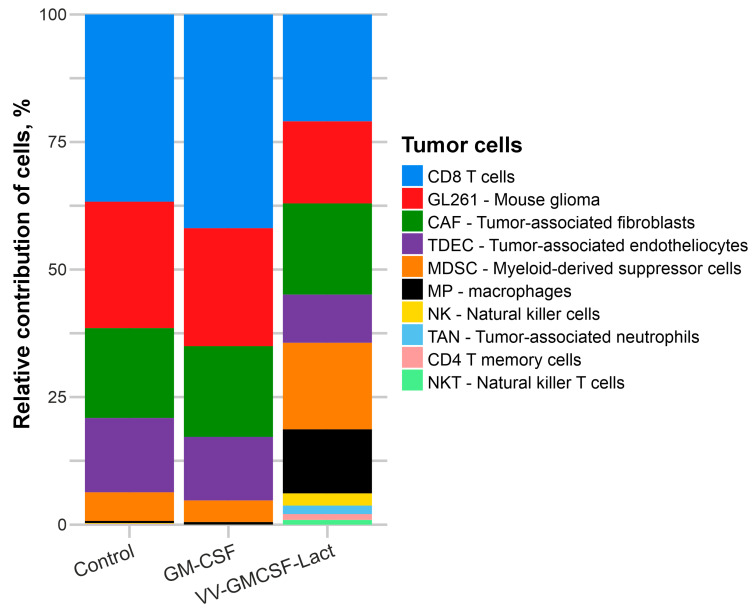
Estimated cell composition of murine glioma GL261 tumors following intratumoral injection of VV-GMCSF-Lact or rhGM-CSF. The interpretation of the designations and the list of SRA cell identifiers are provided in Table S3.

To evaluate changes in the cellular composition of GL261 tumors after VV-GMCSF-Lact or rhGM-CSF administration, transcriptome deconvolution was performed using the unmix function of the DESeq2 package. Deconvolution is a modern bioinformatics approach that estimates the contribution of specific reference datasets to an analyzed array or vector expression profile of experimental data. In this study, transcriptomes from murine GL261 glioma tumors served as the analyzed samples, while a large reference set comprising more than 60 mouse cell transcriptomes from diverse cell types and tissues was used for comparison (Table S2). Reference transcriptomes were compiled from the NCBI SRA database (Table S2). In total, transcriptomes representing over 30 different murine cell types were compared with the GL261 tumor transcriptome. Notably, only 11 cell types contributed significantly (>0.01%) to the tumor transcriptome and were therefore included in subsequent analysis of tumor cellular composition (Figure 10, Table S3).

Data in Table S3 indicate that VV-GMCSF-Lact infection caused a significant (*p* < 0.05) decrease in the contribution of GL261 cells in tumors, from ~25% to ~16%, as well as a significant (*p* < 0.05) decrease in the contribution of CD8^+^ T cells. At the same time, increased contributions of MDSCs, macrophages, NK cells, and TANs were observed. In contrast, rhGM-CSF administration induced only minor changes, limited to the contribution of NK cells (Figure 10, Table S3). These findings, particularly the reduced contribution of CD8^+^ T cells and the increased contributions of MDSCs, macrophages, and TANs, are consistent with the results of differential expression analysis (Figure 9 and Figure 10).

Thus, our data demonstrate that VV-GMCSF-Lact therapy leads to significant remodeling of the TME in murine GL261 gliomas. Flow cytometry analysis showed that the immunotherapeutic effects of the viral treatment targeted distinct components of the murine immune system and differed by animal sex. After VV-GMCSF-Lact administration, we observed a significant reduction in Tregs and monocytic MDSCs in tumors from female mice, while male animals showed a significant decrease in M2-polarized macrophages and CD8^+^ lymphocytes, accompanied by an increase in CD4^+^ lymphocytes. In contrast, rhGM-CSF treatment did not induce comparable changes in the TME and primarily affected immune cells in peripheral organs.

Transcriptome analysis of GL261 gliomas transplanted into mice revealed opposite patterns of gene expression and cellular composition following VV-GMCSF-Lact and rhGM-CSF administration. VV-GMCSF-Lact treatment led to a decrease in the proportion of malignant GL261 cells and CD8^+^ T lymphocytes in tumors, whereas rhGM-CSF treatment was associated with increased proportions of MDSCs, macrophages, NK cells, and TANs.

## 3. Discussion

Gliomas are characterized by an immunosuppressive TME that contributes significantly to tumor growth, progression, and resistance to various therapeutic approaches. In particular, immune cells in the TME act both as an obstacle to therapy and as its potential target. Major populations of such cells include tumor-associated macrophages (TAMs), which elicit T-cell dysfunction and immunosuppression together with myeloid-derived suppressor cells (MDSCs) and T-regulatory lymphocytes (Tregs). As these populations shape the patient’s immune status and therapeutic responsiveness, they represent potential targets for TME sensitization.

Oncolytic viruses are immunotherapeutic agents that can both directly eliminate tumor cells and induce antitumor immune responses, enabling immune-mediated targeting of distant, uninfected tumor cells. In addition, the insertion of immunomodulatory transgenes into the viral genome can help enhance local antitumor immunity. Viral agents also effectively remodel the TME, turning immunologically “cold” tumors into “hot” tumors that are susceptible not only to host immune responses but also to other immunotherapies, such as immune checkpoint inhibitors.

In this study, we first evaluated the secretion of human GM-CSF encoded by VV-GMCSF-Lact in glioma cells of different species following viral infection. Human (immortalized U87MG cells and cells of patient-derived BR3.20 and BR5.21 cultures), murine (immortalized GL261 and CT2A cells), and rat (immortalized C6 cells) glioma cells were infected with VV-GMCSF-Lact, and hGM-CSF levels in the culture medium were measured. While human glioma cells secreted detectable hGM-CSF (Figure 1), no changes in hGM-CSF levels were detected in the culture medium of infected murine or rat glioma cells.

One possible explanation is that hGM-CSF expressed in rodent glioma cells does not undergo appropriate post-translational modifications, resulting in impaired secretion. Cytokines are typically glycosylated through the covalent attachment of mono- and oligosaccharides, which is one of the most common post-translational modifications mediated by complex biosynthetic pathways involving more than 150 glycosyltransferases. hGM-CSF contains two potential N-glycosylation sites and several O-glycosylation sites, and the extent of glycosylation may affect its biological activity, pharmacokinetics, immunogenicity, toxicity, and secretion efficiency [21]. Consistently, site-directed mutagenesis of all N- and O-glycosylation sites reduces transient hGM-CSF secretion compared with the fully glycosylated protein in COS cells [22]. Because the glycosylation sites of hGM-CSF differ significantly from those of rodent GM-CSF (Table S4), hGM-CSF may fail to undergo appropriate modifications in mouse and rat cells or may be modified at non-native sites, leading to decreased secretion. These species-specific differences likely account for the absence of detectable hGM-CSF in the culture medium of virus-infected murine and rat glioma cells.

Importantly, the lack of detectable hGM-CSF secretion in vitro does not preclude its functional activity in syngeneic in vivo models. The main principle of virotherapy is direct tumor cell lysis, resulting in the release of both tumor antigens and virally encoded transgene products into the extracellular space. hGM-CSF released upon tumor cell lysis may therefore recruit immune cells into the TME and stimulate local antitumor immunity. It is known that therapy with recombinant hGM-CSF can elicit immune responses in rodent models [23,24]. Accordingly, the immunostimulatory effects of hGM-CSF expressed by VV-GMCSF-Lact in syngeneic models are likely to manifest after the viral life cycle is completed and tumor cells are lysed.

To elucidate the nature of immunogenic response to the therapy, this study was conducted to examine the immune response of GL261 glioma to both viral and rhGM-CSF therapies. As reflected in Stylli et al. review, the optimal murine glioma model is characterized by invasive and angiogenic tumor growth, cellular heterogeneity, genetic and histologic similarities to human gliomas and an orthotopic location allowing tumor-stroma interactions [25]. Undoubtedly, the orthotopic glioma model is closer to clinical cases; however, for the initial assessment of the immunotherapeutic effect of VV-GMCSF-Lact a more accessible model of subcutaneously transplanted glioma was chosen.

Necrotic foci observed in untreated control tumors are a common feature of large solid tumors, as the tumor core does not receive enough nutrients due to insufficient vascularization during rapid neoplastic growth, leading to localized tumor cell death. Tumor necrosis is often positively correlated with tumor size, aggressiveness, and poor prognosis [26]. The absence of significant differences in tumor size between control and treated groups may reflect extensive immune cell infiltration into the tumor mass.

Vaccinia viruses are known to drive infected cells into S phase, thereby facilitating viral replication while simultaneously suppressing tumor cell proliferation [27]. Indeed, in female mice treated with VV-GMCSF-Lact, tumor cells showed fewer mitotic figures compared with the non-treated control group (Table 2). In male mice, however, no significant differences in mitotic activity were observed between untreated and treated groups, which may be attributed to the presence of stromal cells within the tumor tissue (Figure 2C, Table 2). Stromal cells in the glioma TME are known to be involved in the regulation of tumor proliferation, invasion, angiogenesis, immune response, and, as a consequence, drug resistance [28].

It is important to note that, in male mice, intratumoral administration of rhGM-CSF caused a more extensive tumor tissue destruction than the oncolytic VV-GMCSF-Lact virus treatment, consistent with the observed antitumor efficacy (Figure 2B,C). Overall, histological evaluation of subcutaneously transplanted GL261 tumors revealed that structural changes, including stromal component and baseline mitotic activity, as well as responses to VV-GMCSF-Lact treatment (antitumor effects, extent and nature of tumor tissue damage, and impact on tumor cell proliferation) were sex dependent (Figure 2C, Table 2). These findings align with clinical observations that female patients with gliomas generally respond better to the therapy and have higher survival rates compared to male patients [29].

Since the oncolytic virus VV-GMCSF-Lact functions as an immunotherapeutic agent, it was important to evaluate therapy-induced immune response and compare it with the immunotherapeutic effect of rhGM-CSF. To assess changes in immune status after VV-GMCSF-Lact and rhGM-CSF therapy, blood, tumor tissue, and spleens were collected from C57Bl/6 mice bearing subcutaneously transplanted GL261 gliomas, and immune cell populations were quantified (T and B lymphocytes, Tregs, MDSCs, TAMs).

Immunophenotyping revealed a significant increase in tumor-infiltrating CD4^+^ T-lymphocytes in VV-GMCSF-Lact-treated males compared with controls (Figure 3C). At the same time, no significant changes were observed in Tregs (CD45^+^ CD4^+^ FoxP3^+^) or double-positive (CD45^+^ CD4^+^ CD8^+^) T cells, suggesting a selective expansion of conventional CD4^+^ T helper cells in response to viral therapy. These findings are consistent with a previous study by Grimes et al., which demonstrated that the oncolytic herpes simplex virus M002 increased both the number and frequency of CD4^+^ T helper cells in syngeneic murine GSC005 and GL261-PVRL1 glioma models, resulting in long-term immune memory and enhanced therapeutic efficacy. Notably, depletion of CD4^+^ cells markedly reduced the therapeutic efficacy of the oncolytic virus in that study, whereas CD8^+^ cell depletion had no effect [30].

In female mice, the number of tumor-infiltrating Tregs (CD45^+^ CD4^+^ FoxP3^+^) decreased after both VV-GMCSF-Lact and rhGM-CSF administration (Figure 3D). Given that Tregs are key mediators of immunosuppression in gliomas and limit the effectiveness of immunotherapy, their reduction within the TME suggests a significant therapeutic potential of the oncolytic virus, further enhanced by hGM-CSF expression [31].

Analysis of peripheral T lymphocyte populations showed that, in male mice, VV-GMCSF-Lact therapy led to a decrease in CD3^+^ cells in both blood and spleen compared to the non-treated control group (Figure S2A,B). Such lymphopenia is a common feature of viral infections and is often attributed to immune system activation and lymphocyte redistribution to peripheral tissues [32]. Consistent with this interpretation, Pakola et al. reported that the oncolytic adenovirus TILT-123 elicited an acute decrease in circulating lymphocytes, which correlated with better therapeutic efficacy and patient outcomes [33]. However, cytokine therapy with rhGM-CSF resulted in an increased number of Tregs in the spleen compared to both viral and non-treated control groups, while VV-GMCSF-Lact treatment significantly reduced splenic FoxP3^+^ immunosuppressive T cells relative to untreated animals (Figure S2F). In female mice, VV-GMCSF-Lact treatment was associated with a decrease in splenic CD3^+^ T cells, including CD4^+^ T cells, as well as CD19^+^ B cells, compared to intact animals (Figure S2A,D,E). It is worth noting that the number of CD8^+^ T cells in the spleen increased after oncolytic virus therapy compared to untreated female mice (Figure S2C). Interestingly, therapy with rhGM-CSF also increased splenic CD8^+^ T lymphocyte numbers in both sexes compared with VV-GMCSF-Lact treatment, with a more pronounced effect in females (Figure S2C).

Known for their immunoregulatory function, MDSCs are commonly subdivided into monocytic (m-MDSC) and granulocytic (g-MDSC) populations. However, accurate immunophenotyping of these subsets remains challenging because of their high heterogeneity and the limited specificity of commonly used markers. Current classification relies on the expression of Ly6C and Ly6G. Monocytic MDSCs are typically defined by high Ly6C and low or absent Ly6G expression (Ly6C^high/+^/Ly6G^low/−^), whereas reported marker combinations for granulocytic MDSCs vary (Ly6G^high/+^/Ly6C^+/low/−^) [34,35,36,37]. In several studies, cells expressing Ly6G^high/+^/Ly6C^int/+^ have instead been classified as neutrophils [38,39,40]. Delano et al. reported phenotypical and functional similarities between murine neutrophils induced by polymicrobial sepsis and tumor-associated MDSCs [41]. Accordingly, a practical distinction often applied is that Ly6G expression broadly marks neutrophils or neutrophil-like MDSCs, whereas Ly6C expression identifies monocytes.

In our study, we identified a tumor-infiltrating Ly6C^high^/Ly6G^low^ population consistent with m-MDSC, a subset known for its immunosuppressive role in neoplastic diseases. VV-GMCSF-Lact therapy reduced the number of these cells in tumors from female mice compared with non-treated controls (Figure 3F). By contrast, a distinct population of neutrophilic MDSCs (or g-MDSCs) characterized by high Ly6G and low Ly6C expression was not clearly detected. We therefore speculate that the Ly6C^high^/Ly6G^high^ population observed in our analysis may correspond to neutrophil-like MDSCs, as several studies have reported variable Ly6C expression within this cell type. The abundance of this particular population increased after VV-GMCSF-Lact therapy in both sexes, potentially reflecting emergency granulopoiesis in response to viral infection (Figure 3E) [42].

TAMs play an important role in regulating immune response in neoplastic diseases and are divided into two major subpopulations based on their inflammatory profiles: pro-inflammatory M1 and anti-inflammatory M2 macrophages. In this study, we detected only the M2-polarized anti-inflammatory subset, which is known to promote tumor invasion, metastasis, and resistance to various therapeutic modalities [19]. Despite the greater resistance of tumors in male mice to antitumor therapy, VV-GMCSF-Lact treatment resulted in a decrease in tumor-associated M2 macrophages compared with non-treated controls (Figure 3G).

Overall, our findings demonstrate that oncolytic virotherapy alters the composition of immune cell populations within the TME in a sex-dependent manner. In particular, VV-GMCSF-Lact treatment reduced monocytic MDSCs and Treg lymphocytes in female mice, while selectively decreasing pro-tumor M2 macrophages in male mice. These cell populations contribute significantly to the immunosuppressive environment of gliomas. Viral infection also had a pronounced effect on peripheral lymphocytes, manifesting as lymphopenia, which has been linked to improved clinical outcomes. By contrast, the immunomodulatory effects of cytokine therapy with rhGM-CSF were more limited and manifested mainly as increased numbers of peripheral lymphocytes. Nevertheless, rhGM-CSF treatment induced substantial tumor tissue destruction in male mice.

Although research suggests no cross-species binding between hGM-CSF and the murine GM-CSF receptor (mGMCSF-R) [43], our data indicate pronounced antitumor and immunotherapeutic effects of rhGM-CSF in a murine glioma model. Similar cross-species activity has been reported in other contexts. For example, administration of rhGM-CSF prolonged survival in SARS-CoV-2-infected K18-hACE2 transgenic mice, which are susceptible to viral entry but do not express the hGM-CSF receptor [44]. Therefore, it can be concluded that rhGM-CSF is able to exert biologically relevant effects in mice.

The distinct therapeutic effects observed with VV-GMCSF-Lact and rhGM-CSF treatment were supported by whole-transcriptome analysis of tumor tissue. VV-GMCSF-Lact and rhGM-CSF differentially modulated NFKB-regulated signaling pathways, as shown in Figure 9. Specifically, VV-GMCSF-Lact infection led to significant upregulation of *Ifnb1* and the pro-inflammatory cytokine transcripts Il6, -1B, and Tnf, whereas these transcripts remained unchanged after rhGM-CSF treatment. Additionally, the two therapies had opposing effects in *Ifng* expression. It is known that the cGAS-STING pathway is activated in human cells in response to the presence of double-stranded DNA (dsDNA), which triggers a signaling cascade involving IKK1 and TBK1. These protein kinases activate the transcription factor IRF3, thereby promoting IFNB induction during VV-GMCSF-Lact infection in human cells [19]. In line with this mechanism, mRNA levels of key components of this signaling pathway, including *Sting*, *Ikk1*, and *Tbk1*, were higher during VV-GMCSF-Lact infection (Figure 9).

An important element of immunoregulation within the TME in human cells is the expression of immune checkpoint molecules, particularly PD-L1, which suppresses CD8^+^ T-cell function through interaction with the PD-1 receptor. Increased PD-L1 expression prevents T-cell activation and cytotoxic activity, thereby promoting immunosuppression and immune evasion by malignant cells [45]. On the other hand, the transcription factor BATF plays a key role in the differentiation and function of human CD8^+^ T cells, and has been linked to CD8^+^ T cell exhaustion in chronic inflammation and the TME through regulation of PD-1 and other inhibitory receptors [46,47]. Our data indicate that VV-GMCSF-Lact therapy increases the expression of PD-L1 and BATF, which may contribute to the formation of an immunosuppressive environment that may promote CD8^+^ T-cell depletion, despite the parallel activation of pro-inflammatory processes (discussed in Section 2.4.2). Consistent with this interpretation, flow cytometry revealed a decrease in CD8^+^ T cells in tumors from male mice treated with VV-GMCSF-Lact. In contrast, administration of rhGM-CSF was associated with reduced *BATF* gene expression and no significant change in PD-L1 mRNA levels, indicating a lower immunosuppressive potential and a potential preservation of effector T-cell activity within the tumor (Figure 9). Nevertheless, these transcriptional differences were not reflected in detectable changes at the cellular level by flow cytometry.

VV-GMCSF-Lact infection also upregulated chemokines involved in immune cell recruitment. In particular, transcriptome analysis indicated activation of pathways associated with neutrophil chemotaxis, consistent with the observed increase in the Ly6C^high^/Ly6G^high^ MDSC population (Figure 7A). Moreover, rhGM-CSF therapy was associated with downregulation of *Ccl5*, a chemokine implicated in MDSC recruitment, as well as *Ccl4* and *Ccl3*, which are linked to monocyte chemotaxis in human cells (Figure 8B). These findings suggest a dual role of rhGM-CSF in immunomodulation [48,49].

Genes encoding anti-apoptotic proteins, including *Gadd*45 and *Bcl2a1* [50,51], exhibited opposing expression patterns between treatments, with increased expression following VV-GMCSF-Lact infection and decreased expression after rhGM-CSF administration. These results may indicate that VV-GMCSF-Lact and rhGM-CSF differentially modulate apoptotic pathways in GL261 tumor cells transplanted into mice (Figure 9).

According to the obtained transcriptome analysis results, VV-GMCSF-Lact infection activated the transcription factors NFKB1/RELA and SMAD4, together with their associated signaling pathways, pro-inflammatory responses, and immune activation. On the contrary, rhGM-CSF treatment was accompanied by suppression of these transcription factors and inhibition of immune-related processes.

Another important determinant of virotherapy efficacy is the extracellular matrix (ECM), a key structural and functional component of the TME. ECM can restrict the spread of viral particles within the tumor and limit immune cell infiltration [52]. In our study, viral therapy with VV-GMCSF-Lact downregulated multiple collagens and cadherins (Figure 7B). Computational modeling by Pooladvand and Kim demonstrated that high collagen density impairs viral diffusion [53], suggesting that ECM may directly impact virotherapy efficacy, limiting the dissemination of newly reproducing viral particles to surrounding tumor cells and distant nodules and metastases. Collagens are also known to promote glioma cell proliferation, migration, and invasion, thereby facilitating tumor progression [54]. Protogadherin-10, the expression level of which was also reduced according to our data, has been implicated as an oncogene in gliomas, contributing to tumor cell proliferation and migration [55,56]. Thus, VV-GMCSF-Lact therapy may reduce glioma cell proliferation and motility, thereby improving therapeutic outcomes.

## 4. Materials and Methods

### 4.1. Glioma Cell Cultures

Rat C6 glioma cells (ATCC CCL-107) were obtained from the Collection of Cell Cultures of the Federal Research and Clinical Center of the Federal Medical-Biological Agency (Moscow, Russia) and cultured in IMDM medium (Thermo Fisher Scientific, Waltham, MA, USA) supplemented with 10% FBS (Thermo Fisher Scientific, Waltham, MA, USA), 2 mM L-glutamine (Invitrogen, Waltham, MA, USA), and antimycotic/antibiotics solution (100 U/mL penicillin, 100 mg/mL streptomycin sulfate, 0.25 μg/mL amphotericin; Sigma-Aldrich, Darmstadt, Germany) in 25 cm^2^ culture flasks (TPP, Trasadingen, Switzerland).

Murine GL261 (DSMZ ACC-802) and CT2A (Sigma SCC-194) glioma cells, kindly provided by Alexey Stepanenko (Department of Neurobiology, Serbsky National Medical Research Center for Psychiatry and Narcology, Moscow, Russia) were cultured in DMEM (Thermo Fisher Scientific, Waltham, MA, USA) supplemented with 10% FBS (Thermo Fisher Scientific, Waltham, MA, USA), 2 mM L-glutamine (Invitrogen, Waltham, MA, USA), and antimycotic/antibiotics solution (100 U/mL penicillin, 100 mg/mL streptomycin sulfate, 0.25 μg/mL amphotericin; Sigma-Aldrich, Darmstadt, Germany) in 25 cm^2^ culture flasks (TPP, Trasadingen, Switzerland).

Patient-derived glioma tissue samples were obtained at the Novosibirsk Research Institute of Traumatology and Orthopedics (Novosibirsk, Russia) as described previously [57]. The BR3.20 and BR5.21 patient-derived glioma cells were cultured in 6-well plates using IMDM supplemented with 10% FBS, 2 mM L-glutamine, 100 U/mL penicillin, 100 µg/mL streptomycin, and 250 mg/mL amphotericin B.

All cells were incubated at 37 °C in a humidified atmosphere containing 5% CO_2_ for cell adhesion.

### 4.2. Analysis of VV-GMCSF-Lact-Induced GM-CSF Secretion by Glioma Cells

Human U87MG, BR3.20, and BR5.21 glioma cells, murine GL261 and CT2A glioma cells, and rat C6 glioma cells were seeded into 6-well plates at a density of 5 × 10^5^ cells per well. After the formation of a cell monolayer, the cell medium was collected, followed by the addition of 500 μL of nutrient medium containing VV-GMCSF-Lact at a multiplicity of infection (MOI) of 0.1 PFU/cell. Infected cells were placed in an incubator for 1 h, after which 1 mL of the nutrient medium was added to each well. After 24 and 48 h from the beginning of incubation with the virus, the growth medium was collected for further analysis by ELISA using a commercial human GM-CSF detection kit (Colony Stimulating Factor 2, Granulocyte Macrophage ELISA Kit; ELK Biotechnology, Sugar Land, TX, USA). Uninfected cells were used as a control. Three biological replicates were made at each time point.

### 4.3. Experimental Animals

All animal procedures were carried out at the Institute of Chemical Biology and Fundamental Medicine, Siberian Branch of the Russian Academy of Sciences (SB RAS; Novosibirsk, Russia). The study was carried out in compliance with the ARRIVE guidelines. All experiments with laboratory animals were carried out in accordance with the recommendations and requirements of the World Society for the Protection of Animals (WSPA) and the European Convention for the Protection of Experimental Animals (Strasbourg, 1986) [58]. The protocol was approved by the Bioethics Committee of the Institute of Cytology and Genetics SB RAS (protocol No 197 dated 21 November 2024).

The study was performed in 27 male and 30 female C57Bl/6 mice (*n* = 57; body weight 18–20 g) obtained from the SPF Vivarium of the Institute of Cytology and Genetics SB RAS (Novosibirsk, Russia). Mice were housed in groups in standard cages under a 12 h light/dark cycle, at a temperature of 20–24 °C and a humidity of 45–55%. Water and standard chow were provided ad libitum.

### 4.4. Subcutaneous Transplantation of GL261 Glioma Cells and Tumor Treatment

To induce subcutaneous tumors, 3 × 10^6^ GL261 cells in 100 μL of saline and Matrigel Matrix (Corning, Corning, NY, USA) mixed in a 1:1 *v*/*v* ratio were transplanted into the back area of 27 male and 30 female C57Bl/6 mice (*n* = 57). Tumor development was monitored by measuring the linear size of tumors with a caliper. When the tumors were formed and reached a size of 120–150 mm^3^, mice were randomized into 3 groups, 10 females and 9 males each, using the = RAND() function in Microsoft Excel v.16 (Redmond, WA, USA) (Table 3). An intact control was also used (3 mice of each sex).

Each mouse was considered an experimental unit. The sample size was calculated using Lamorte’s Power Calculations (https://www.bu.edu/research/forms-policies/iacuc-sample-size-calculations/ accessed on 12 June 2024). Mice were included in the experiment if a tumor node was present at the transplantation site and the animal was healthy at therapy onset. Exclusion criteria were the absence of a tumor node (for all mice that underwent tumor cell transplantation) or poor health. No animals met these exclusion criteria, and all 57 mice were included in the experiment. Treatments were administered according to Table 3, and the order of treatment and tumor volume measurements was randomized daily.

Investigators involved in animal treatment were aware of group allocation. Investigators performing immune cell isolation, flow cytometry, RNA extraction, and transcriptome analysis were blinded to group allocation.

Primary outcomes of this study were tumor size, the proportions of immune cell populations in tumors, spleen, and blood, and tumor transcriptome profiles. Secondary outcomes included morphological changes in tumor tissue.

### 4.5. Immune Status of Mice with Subcutaneously Transplanted GL261 Glioma During Therapy with VV-GMCSF-Lact

Blood, tumor tissue, and spleens were collected from mice with subcutaneously transplanted GL261 gliomas to assess the changes in immune status (T cells, B cells, myeloid cells and/or macrophages) following antitumor therapy with VV-GMCSF-Lact. Immune blood cells were isolated using Ficoll-Urografin solution with a density of 1.077 g/cm^3^. Cells were then labeled with mouse antibodies against CD3 conjugated with FITC (Invitrogen, Waltham, MA, USA), CD4 conjugated with APC (Invitrogen, Waltham, MA, USA), CD45.2 conjugated with PE-Cy7 (Elabscience, Houston, TX, USA), CD8a conjugated with Super Bright 436 (Invitrogen, Waltham, MA, USA), FOXP3 conjugated with PE (Invitrogen, Waltham, MA, USA), and CD19.

The spleens were mechanically dissociated and splenocytes were stained using FITC-conjugated mouse anti-CD3 antibodies (Invitrogen, Waltham, MA, USA), APC-conjugated mouse anti-CD4 antibodies (Invitrogen, Waltham, MA, USA), PE-Cy7-conjugated mouse anti-CD45.2 antibodies (Elabscience, Houston, TX, USA), Super Bright 436-conjugated mouse anti-CD8a antibodies (Invitrogen, Waltham, MA, USA), PE-conjugated mouse anti-FOXP3 antibodies (Invitrogen, Waltham, MA, USA), and CD19.

Tumor tissue was dissociated mechanically and enzymatically using collagenase IV and DNase I. Immune cells from tumor homogenates were isolated using Ficoll-Urografin solution in a density gradient of 1.077 g/cm^3^. Then, the cells were stained using mouse antibodies against CD3 conjugated with FITC (Invitrogen, Waltham, MA, USA), CD4 conjugated with APC (Invitrogen, Waltham, MA, USA), CD45.2 conjugated with PE-Cy7 (Elabscience, Houston, TX, USA), CD8a conjugated with Super Bright 436 (Invitrogen, Waltham, MA, USA), FOXP3 conjugated with PE (Invitrogen, Waltham, MA, USA), CD19 conjugated with APC (Sony Biotechnology, San Jose, CA, USA), CD206 conjugated with FITC (Invitrogen, Waltham, MA, USA), CD86 (B7-2), conjugated with APC (Invitrogen, Waltham, MA, USA), F4/80 conjugated with PE (Invitrogen, Waltham, MA, USA), CD11b conjugated with PE-Cy7 (Sony Biotechnology, San Jose, CA, USA), Ly6C conjugated with APC (Invitrogen, Waltham, MA, USA), Ly6G conjugated with FITC (Invitrogen, Waltham, MA, USA). All samples were analyzed using a FACSCantoII flow cytometer. Data analysis was performed using FACSDiva software version 2.4 (BD Biosciences, Franklin Lakes, NJ, USA). The gating strategy is presented in Figure S4.

### 4.6. Histology and Morphometry

For histological analysis, tumor specimens were fixed in 10% neutral-buffered formalin (BioVitrum, Moscow, Russia), dehydrated in ascending concentrations of ethanol and xylene, and embedded in HISTOMIX paraffin (BioVitrum, Moscow, Russia). Paraffin sections (up to 5 μm) were cut using a Microm HM 355S microtome (Thermo Fisher Scientific, Waltham, MA, USA) and stained with hematoxylin and eosin. Images were captured and examined using an Axiostar Plus microscope equipped with an Axiocam MRc5 digital camera (Zeiss, Oberkochen, Germany) at ×200 magnification.

Morphometric analysis of tumor nodules was performed using a closed test system with a test area of 3.2 × 10^6^ μm^2^ and included evaluation of numerical density (Nv) of mitotic figures, defined as the number of cells undergoing mitosis within the test area. Ten random fields from tumor specimens of three to five mice in each group were analyzed (30–50 fields in total).

### 4.7. RNA Sequencing

Illumina cDNA libraries were produced according to a standard protocol using the NEBNext Ultra II Directional RNA Library Prep Kit for Illumina (New England Biolabs, Ipswich, MA, USA) and the NEBNext Poly(A) mRNA Magnetic Isolation Module (New England Biolabs, Ipswich, MA, USA), as well as by mass parallel sequencing on FastaSeq300 (GeneMind, Shenzhen, China) at the Institute of Fundamental Medicine and Biology of the Kazan Federal University (Kazan, Russia). RNA integrity was assessed using a Bioanalyzer 2100 system (Agilent, Santa Clara, CA, USA) and the Agilent RNA Pico Kit (Agilent, Santa Clara, CA, USA). For library preparation, 500 ng of total tumor RNA was used. Fragment size distribution in the sequencing libraries was analyzed using Bioanalyzer 2100 (Agilent, Santa Clara, CA, USA) with the Agilent High Sensitivity DNA Kit (Agilent, Santa Clara, CA, USA). The libraries were quantified using a Qubit 2.0 Fluorometer (Invitrogen, Waltham, MA, USA) with the Qubit dsDNA HS Assay Kit (Thermo Fisher Scientific, USA). The FASTASeq 300 V2.0 Kit (GeneMind, Shenzhen, China), producing 75-nucleotide single reads, was used for sequencing.

### 4.8. Transcriptome Analysis

Primary sequencing data, consisting of nucleotide sequences in FASTQ format, were aligned to the mouse and VV-GMCSF-Lact genomes using STAR v2.7.1a. For this purpose, we assembled a composite genome that included mouse genomic sequences (GRCm39/mm39 assemblies) and the VV-GMCSF-Lact viral genome as a separate chromosome, together with the corresponding transcript annotations in GTF format. Transcript-level contributions were quantified by STAR v2.7.1a during the alignment process.

Transcriptome analysis of GL261 glioma tumors subcutaneously transplanted into C57Bl/6 mice was performed using R v4.1.3 and Bioconductor v3.14. Principal component analysis (PCA) was conducted using the prcomp function from base R with default parameters, and results were visualized with ggplot2 v3.5.1. For hierarchical clustering, pairwise distances between samples were calculated using the Euclidean metric with the dist function (base R, default settings). Clustering was performed with the hclust function (base R), and dendrograms were visualized using the base R plot function.

Differential gene expression analysis was performed using DESeq2 1.36.0, R version 4.1.3, and Bioconductor 3.14. Following differential gene expression analysis, lists of upregulated and downregulated genes were analyzed using Enrichr via the R package enrichR (v3.4) [20].

To assess changes in cellular composition of transplanted GL261 glioma tumors following VV-GMCSF-Lact or rhGM-CSF administration, the unmix function from the DeSeq2 package was used. A representative set of murine reference transcriptomes was assembled from the NCBI Sequence Read Archive (SRA). RNA-seq data in FASTQ format from various murine cells, organs, and tissues were aligned to both the mouse and VV-GMCSF-Lact genomes using STAR v2.7.1a. Transcript-level contributions for each dataset were quantified, and relative contributions of murine reference transcriptomes to the GL261 tumor transcriptome were subsequently analyzed.

### 4.9. Statistics

Significance was determined using a two-tailed Student’s *t*-test. Error bars represent the standard deviation of the mean. A *p*-value < 0.05 was considered statistically significant.

## 5. Conclusions

The development of new approaches to glioma therapy remains a pressing challenge in modern oncology. At the same time, virotherapy is emerging as one of the most actively explored treatment strategies. Despite high oncoselectivity and the capacity not only to destroy tumor cells directly but also to engage antitumor immunity, oncolytic viruses can have opposing immune effects. A clearer understanding of oncolytic virus activity, therefore, requires detailed investigation of both their mechanism of action and their contribution to modulation of the TME, particularly immune components.

The main goal of this study was to compare the antitumor immune effects of the oncolytic vaccinia virus VV-GMCSF-Lact with those induced by recombinant human GM-CSF in human and rodent glioma models and in immunocompetent mice bearing GL261 gliomas. VV-GMCSF-Lact activated antitumor immune responses irrespective of animal sex, yet distinct immune components were differentially involved in male and female animals. Moreover, virus therapy activated transcription factors that mediate signaling pathways associated with inflammation, cytokine and interferon signaling, and neutrophil chemotaxis, while downregulating expression of genes encoding extracellular matrix protein.

Taken together, these findings show that VV-GMCSF-Lact reshapes the glioma immune microenvironment through sex-dependent depletion of key immunosuppressive populations and activation of inflammatory and antiviral pathways, whereas rhGM-CSF exerts distinct and more limited immunomodulatory effects. The induction of immune checkpoint-associated transcripts during viral therapy supports the rationale for combining VV-GMCSF-Lact with checkpoint blockade or other strategies that prevent adaptive immune resistance.

## Figures and Tables

**Table 1:** Oncolytic activity of VV-GMCSF-Lact against human glioma cells.

Cell Culture	CD50 (PFU/Cell)
BR3.20	0.02 ± 0.0015
BR5.21	0.5 ± 0.018
U87MG	0.1 ± 0.028

CD50—multiplicity of viral infection causing 50% cell death.

**Table 2:** Mitotic activity of subcutaneously transplanted murine GL261 gliomas in C57Bl/6 mice after intratumoral administration of VV-GMCSF-Lact or rhGM-CSF and in untreated controls.

	Mitotic Cell Density, Nv	
	Female	Male
Control group	7.9 ± 0.2	2.7 ± 0.6
VV-GMCSF-Lact	5.5 ± 0.8 *	3.2 ± 0.2
rhGM-CSF	-	3.1 ± 0.2

Nv, the numerical density value, is defined as the number of cells in mitosis (mitotic figures) per test area. Differences are statistically significant compared with the control group at *p* ≤ 0.05 (*).

**Table 3:** Experimental scheme for evaluating VV-GMCSF-Lact and rhGM-CSF immunotherapy against GL261 murine glioma in vivo.

Group	Dose	Administration Route	Administration Scheme
VV-GMCSF-Lact	3.5 × 10^7^ PFU/mouse	Intratumoral	1 injection every 7 days (3-week course)
rhGMCSF	10 µg per kg	Intratumoral	2 injections over 2 days in a row, every 7 days (3-week course)
Saline (non-treated control group)	-	Intratumoral	1 injection every 7 days (3-week course)
Intact	-	-	-

## Data Availability

The original contributions presented in this study are included in the article/Supplementary Material. Further inquiries can be directed to the corresponding authors.

## Supplementary Materials

The following supporting information can be downloaded at: https://www.mdpi.com/article/10.3390/ph19030434/s1, Figure S1: Western blot analysis of hGM-CSF expression in mouse and rat glioma cells infected with VV-GMCSF-Lact; Figure S2: Peripheral CD3^+^ (A), CD8^+^ (C), CD19^+^ (D), CD4^+^ (E), FoxP3^+^ (F) splenic T and B lymphocyte populations and blood CD3^+^ (B) T-cell population in subcutaneously transplanted GL261 tumors after VV-GMCSF-Lact and rhGMCSF therapy. Values represent the mean ± SD of three independent experiments (* *p* ≤ 0.05, ** *p* ≤ 0.01, *** *p* ≤ 0.001); Table S1: Decoding of GL261 tumor sample designations and number of polyA-RNA cDNA reads. PE—paired ends, SE—single ends; Figure S3: Heat maps of gene expression changes in GL261 tumors transplanted into C57Bl/6 mice following intratumoral injections of rhGM-CSF. Normalized expression levels of top 50 transcripts increased (Up) or decreased (Down) after rhGM-CSF treatment are shown; Table S2: SRA murine transcriptomic data used as reference datasets for deconvolution analysis; Table S3: Changes in cellular composition of subcutaneously transplanted GL261 tumors following intratumoral injections of VV-GMCSF-Lact or rhGM-CSF; Table S4: Potential glycosylation sites of human (hGM-CSF), murine (mGM-CSF), and rat (rGM-CSF) GM-CSF (UniProt https://www.uniprot.org/); Figure S4: Gating strategies used to identify specific immune cell subpopulations in tumors, spleens and blood of tumor-bearing and intact C57Bl/6 mice. A. T- and B-cell; B. Tregs; C. MDSC; D. Macrophages.
