# Supplementary material for: Oncolytic Virus VV-GMCSF-Lact and Human GM-CSF Against GL261 Glioma in Immunocompetent Mice
Source: Pharmaceuticals (Basel). 2026 Mar 6;19(3):434. doi: 10.3390/ph19030434 (PMC13029571; doi:10.3390/ph19030434)
Supplement: Supplementary file 1 [file pharmaceuticals-19-00434-s001.zip › pharmaceuticals-4141108-supplementary.pdf]

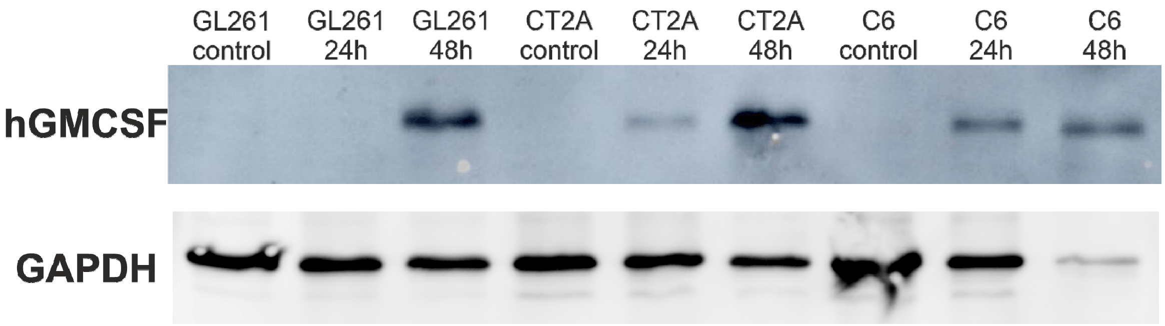

Figure S1. Western Blot analysis of hGM-CSF expression in mouse and rat glioma cells infected with VV-GMCSF-Lact.

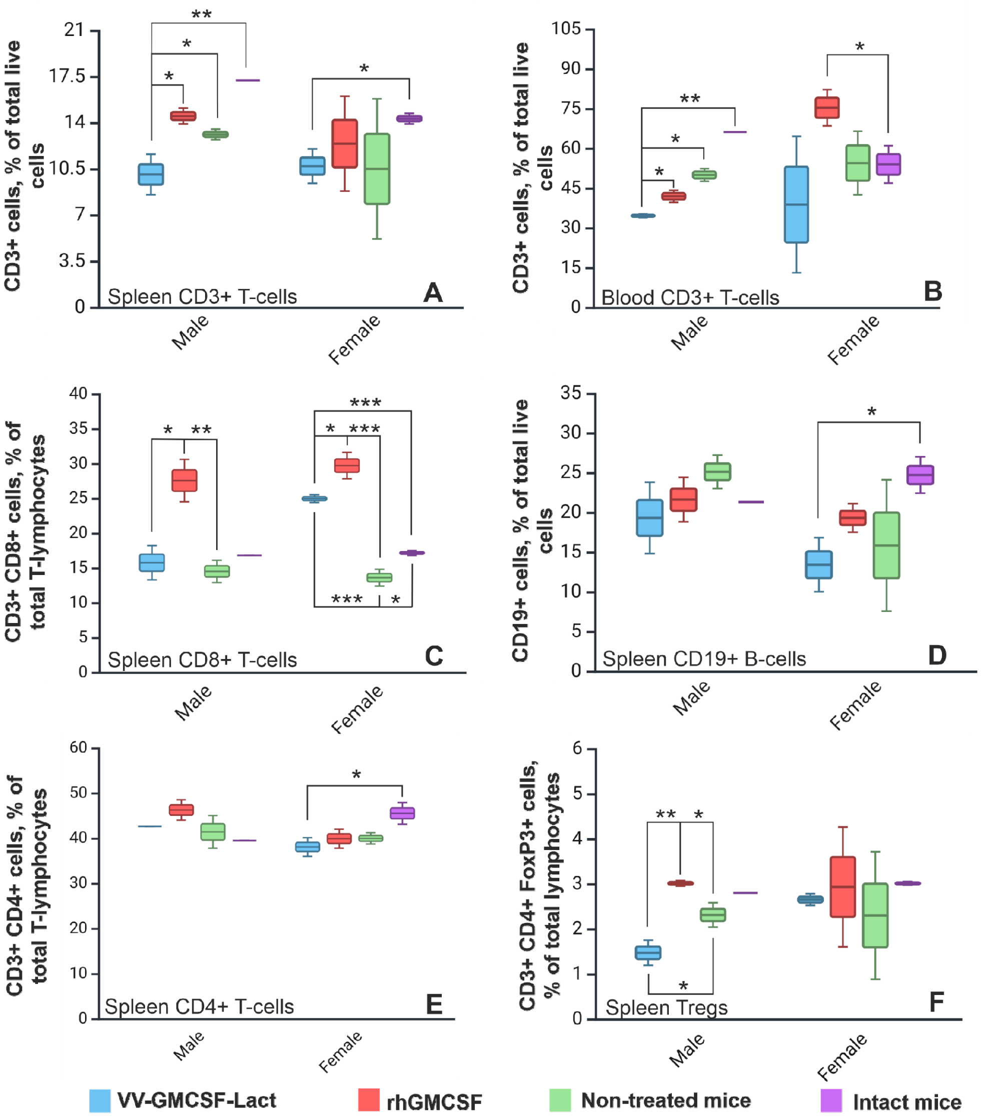

Figure S2. Peripheral CD3^{+} (**A**), CD8^{+} (**C**), CD19^{+} (**D**), CD4^{+} (**E**), FoxP3^{+} (**F**) splenic T and B lymphocyte populations and blood CD3^{+} (**B**) T-cell population in subcutaneously transplanted GL261 tumors after VV-GMCSF-Lact and rhGMCSF therapy. Values represent the mean ± SD of three independent experiments (*p≤0,05, **p≤0,01, ***p≤0,001).

Table S1. Decoding of GL261 tumor sample designations and the number of polyA-RNA cDNA reads. PE – paired ends, SE – single ends.

Sample	Sex	Description	Number of reads	Sequencing read types
Cf05	Female	Control GL261 tumors	30 500 755	PE
Cf08	Female		28 162 141	PE
Cm11	Male		31 345 166	PE
Cm14	Male		28 658 417	PE
hGf04	Female	Tumors injected with recombinant human GMCSF	29 198 903	PE
hGf06	Female		26 216 924	PE
hGm12	Male		17 082 133	SE
hGm13	Male		14 637 124	SE
Vf01	Female	Tumors infected with VV-GMCSF-Lact	28 294 024	PE
Vf02	Female		29 428 627	PE
Vm15	Male		27 013 202	PE
Vm16	Male		28 439 077	PE

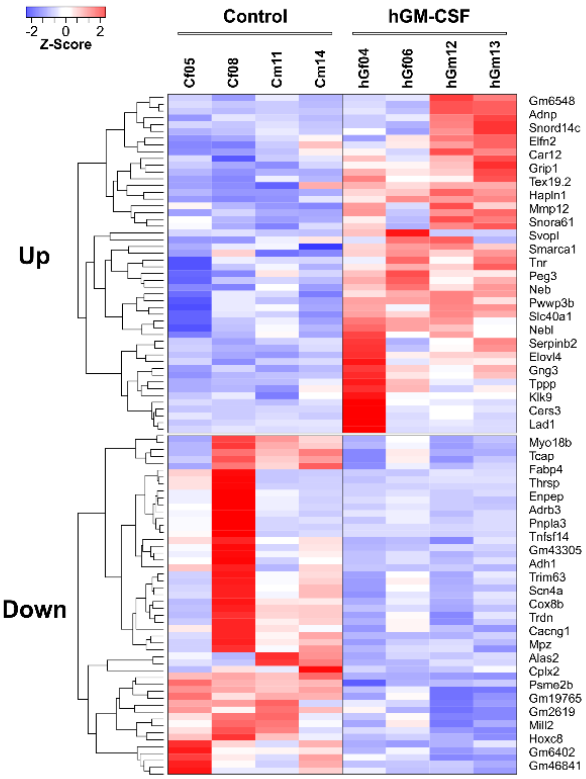

Figure S3. Heat maps of gene expression changes in GL261 tumors transplanted into C57Bl/6 mice upon injections with rhGMCSF. Normalized expression levels of top 50 transcripts increased (Up) or decreased (Down) by intratumor rhGMCSF injections are shown.

Table S2. SRA murine transcriptomic data used as the references for deconvolution analysis.

Sample	Cell type/Tissue type*
GSM8377205	Adipocytes
GSM8377210	B-cells
GSM8377222	Beta cells
GSM2453449	Brain
GSM2453450	Brain
GSM8377229	Brain endothelial
GSM7885393	Primary CAF
GSM7106656	CAF
GSM8377239	CD4 Memory
GSM8377246	CD4 Naive
GSM8430295	CD4+ T Cells
GSM8377252	CD8 Memory
GSM8377259	CD8 Naive
GSM8377269	CD8 Virtual Memory
GSM8561399	T cells
GSM5528232	GL261
GSM5528233	GL261
GSM8377286	Hematopoietic stem cells
GSM8377294	Keratinocytes
GSM8377318	Lymph endothelial
GSM8377326	Lymph fibroblasts
SRX26219313	Macrophages**
SRX26219314	Macrophages**
SRX26219315	Macrophages**
GSM7666312	Myeloid-derived suppresor cells
GSM7474811	Myeloid-derived suppresor cells
GSM8377332	Muscle fibroblasts
GSM8375837	NK cells
GSM8375838	NK cells
GSM6225718	iNKT cell
GSM7517708	Immune cells in the Melanoma
GSM8344661	Natural killer T cells
GSM8377343	Satellite stem cells
GSM8073311	Dorsal skin
GSM8073312	Dorsal skin
GSM8073313	Dorsal skin
GSM8377349	Skin fibroblasts
ERX10010770	Neutrophils**
GSM6672279	Neutrophils
GSM6754057	Primary cells
GSM7321008	Primary immune: neutrophils
GSM4808297	Tumor derived endothelial cells
GSM5974192	TDEC
GSM8377358	Thymic epithelial
GSM8377367	Thymic fibroblasts
GSM8548420	Regulatory T cell

GSM8020816	CD4+CD25+ Treg
GSM8245724	Treg

* – “Cell type” or “Tissue type” fields from “Common Fields” SRA data.
** – Sample description.

Table S3. Changes in the cellular composition of subcutaneously transplanted GL261 tumors upon intratumoral injections of VV-GMCSF-Lact or rhGMCSF.

SRA ID	Description	Average, %			T-test *p**	
		Control	rhGMCSF	VV-GMCSF-Lact	Control vs rhGMCSF	Control vs VV-GMCSF-Lact
GSM8561399	CD8 T cells	36.7	41.9	21.0	0.109	0.033
GSM5528233	murine glioma GL261	24.8	23.1	16.1	0.306	0.027
GSM7106656	CAF tumor-associated fibroblasts	17.6	17.8	17.8	0.479	0.468
GSM5974192	TDEC tumor associated endotheliocytes	14.6	12.5	9.50	0.251	0.160
GSM7474811	MDSC myeloid-derived suppressor cells	5.60	04.2	17.0	0.135	0.0005
SRX26219313	Macrophages	0.40	0.50	12.6	0.311	0.005
GSM8375837	NK natural killer cells	0.10	0.00	2.40	0.020	0.047
ERX10010770	TAN neutrophils associated with tumor	0.00	0.00	1.70	0.196	0.043
GSM8377239	CD4 T memory cells	0.00	0.00	1.10	NA	0.092
GSM7517708	NKT natural killer T cells	0.00	0.00	0.90	NA	0.092
GSM8245724	Treg regulatory T cells	0.20	0.00	0.00	0.196	0.196

* – significance level, T-test. Values of $p < 0.05$ are highlighted in red.

Table S4. Potential glycosylation sites of human (hGM-CSF), murine (mGM-CSF) and rat (rGM-CSF) GM-CSF (UniProt https://www.uniprot.org/).

Type of glycosylation	Glycosylation site		
	hGMCSF	mGMCSF	rGMCSF
O-glycosylation	Ser-22	Ser-22	Ser-22
	Ser-24	Thr-27	Thr-27
	Ser-26		
	Ser-27		
N-glycosylation	Asn-44	Asn-83	Asn-86
	Asn-54	Asn-92	

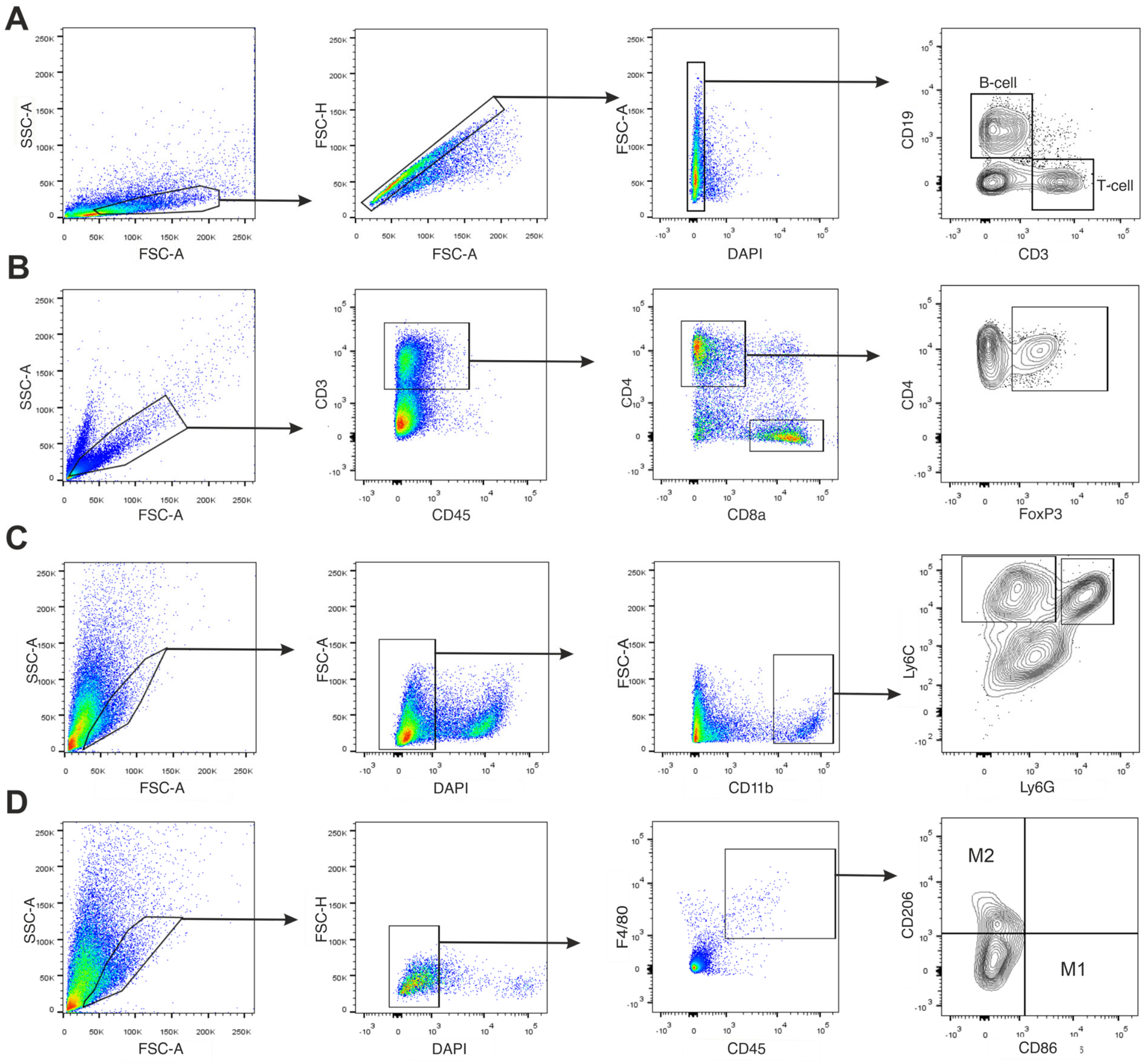

Figure S4. Gating strategies used to identify specific immune cell subpopulations in tumors, spleens and blood of tumor-bearing and intact C57Bl/6 mice. **A.** T- and B-cell; **B.** Tregs; **C.** MDSC; **D.** Macrophages.
